# Supplementary material for: Changeover from signalling to energy-provisioning lipids during transition from colostrum to mature milk in the giant panda (Ailuropoda melanoleuca)
Source: Sci Rep. 2016 Nov 3;6:36141. doi: 10.1038/srep36141 (PMC5093549; doi:10.1038/srep36141)
Supplement: Supplementary Information [file srep36141-s1.pdf]

Changeover from signalling to energy-provisioning lipids during transition from colostrum to mature milk in the giant panda (*Ailuropoda melanoleuca*)

Supplementary Information

Tong Zhang*[§1], David G. Watson[1], Rong Zhang[1,2], Rong Hou[3], I. Kati Loeffler[3#], and Malcolm W. Kennedy[4]**

[1]Strathclyde Institute of Pharmacy and Biomedical Sciences, 161, Cathedral Street, Glasgow G4 0RE, Scotland, UK.

[2]Institute of Clinical Pharmacology, Guangzhou University of Chinese Medicine, No. 12 Jichang Road, Guangzhou 510405, P.R. China.

[3]Sichuan Key Laboratory of Conservation Biology for Endangered Wildlife, Chengdu Research Base of Giant Panda Breeding, 1375 Panda Road, Northern Suburb, Chengdu, Sichuan Province 610081, P.R. China.

[4]Institute of Biodiversity, Animal Health and Comparative Medicine, and Institute of Molecular Cell and Systems Biology, College of Medical, Veterinary, and Life Sciences, Graham Kerr Building, University of Glasgow, Glasgow G12 8QQ, Scotland, UK.

[§] Current address: Institute of Cancer Sciences, Wolfson Wohl Translational Cancer Research Centre, Garscube Estate, Switchback Road, Glasgow G61 1QH, UK.

[#] Current address: International Fund for Animal Welfare, 290 Summer St., Yarmouth Port, MA 02675, USA

*Corresponding author (lipidomics): Tong Zhang, tong.zhang@glasgow.ac.uk.

**Corresponding author (lactation biology): Malcolm Kennedy, malcolm.kennedy@glasgow.ac.uk

Contents

Panel S1. Detailed structural determination of lipids – details additional to Materials and Methods explained in main text.

Table S1. Predominant lipids in giant panda milk.

Figure S1 – Time courses for selected lipids in the milk of three giant pandas with time after birth.

Figure S2 – Changes in appearance of giant panda milk with time.

Figure S3 – Comparison between the time-dependent lipid profiles of giant panda milk with lipid content of artificial milk replacers.

Figure S4 – Mass spectra of the main lipids identified in giant panda milk.

Table S2. Reproductive histories and milk sampling times of giant pandas from whom samples were collected.

References for Supplementary Information.

Note – all the data obtained in this study are available from the corresponding author.

Panel 1. Detailed structural determination of lipids – details additional to Materials and Methods explained in main text

Following the procedures detailed in the main text, the pre-processed lipid-chromatography-mass spectrometry (LC-MS) data were searched by accurate mass (±3 ppm) against the Lipid Metabolites and Pathways Strategy (Lipid MAPS) Structure Database in order to focus on lipid analysis. For annotation of glycerolphospholipids including phosphatidylcholine (PC), phosphatidylethanolamine (PE), phosphatidylserine (PS) and phosphatidylinositol (PI), a well-defined formula prediction algorithm in MZMine 2.10 used previously [1] was also applied. The LC-MS signals were removed or ignored if their coefficient of variation values were ≥ 25% in three separate quality control injections (YY-19d). Only a single LC-MS signal for each lipid was retained if the lipid was detected in both electrospray ionisation (ESI) positive and negative modes at the same retention time. Because the LC-MS experiments were performed in hydrophilic interaction liquid chromatography mode, polar lipids including PC, PE, PS, PI and sphingomyelin were well-retained on the silica column and separated according to their polar head group classes, whereas the non- or low-polarity lipids eluted quite early without sufficient retention. As reported in previous studies of LC-MS-based lipid analysis, some classes of lipids could be detected in both ESI-positive and -negative modes as forms of non-proton adduct ions (e.g. ammonium and formate adducts). As shown in Table S1, selection of the correct adduct forms in different ESI modes for accurate mass search of each lipid class can avoid confusion in putative annotation. Glycerolphospholipids with a single acyl tail showed slightly longer retention times than those with the same polar head but double acyl tails, which may be explained by their slight increase of hydrophilicity due to the presence of an exposed hydroxyl group in the glycerol backbone structure.

Further lipid structure elucidation was performed using collision-induced dissociation multiple stage mass spectrometry (MS^n) fragmentation in a data-dependent scan mode. Different lipid classes could be identified by their characteristic MS^2 fragmentation patterns representing the loss of the specific polar heads (Table S1). The fragment ions produced by losing a water molecule equivalent could be included with the phospholipids in ESI-positive mode if the phospholipids have a free hydroxyl group in their backbone structures. MS^3 fragmentation, especially in ESI-negative mode, provided more details in structural information of the acyl tails. An example of this is given in Figure S4, depicting the MS^2 product ion of a PS that was further fragmented. The resulting MS^3 fragment ions indicate the number of carbons of each acyl tail with the corresponding degree of unsaturation (18:0/22:6). In addition, the relative intensity of the fatty acid ions might imply the position of the acyl tail (*sn*-1 > *sn*-2). Consequently, as shown in Figure S3, the acyl tail of 18:0 was

assigned at *sn*-1 position and the 22:6 was at *sn*-2. More examples of MS^n spectra for the other lipid classes are shown in Figure S4.

The MS^n spectra of polar lipids obtained in this study accorded with the published data produced for Thermo Scientific mass spectrometers (LTQ ion trap or LTQ-Orbitrap) by the manufacturer. Analysis of MS^n spectra for each lipid class or subclass revealed that the abundant polar lipids in giant panda milk possessed acyl chains comprising even numbers of carbons from 14 to 26 with the number of double bonds varying from 0 to 6. However, acyl chains with odd numbers of carbons were also found on both mono and diacyl glycerolphospholipids but were present in low relative abundance. Compared to a single phospholipid species, a single triacylglyceride lipid species generated more fragment ions through the loss of heterogeneous fatty acid tails in the population of molecules (M-RCOO+H), indicating the presence of a more extensive range of isomers in the neutral than in the polar lipids.

Another explanation for the apparently low abundance of glycerolipids may lie in the interpretation of the low MS signal for this group of molecules. Glycerolipids provide low-abundance MS signals because of their poor ESI performance such that the intensity of their MS signals did not provide quite the same clarity in changes in their relative concentrations in comparison with the polar lipids, despite being the major lipids in mammalian milks including that of giant panda.

In the data-dependent scan mode, the MS^n fragmentation was only triggered on those lipids with the highest intensities in the MS^1 scans. Interpretation of the MS^n spectra suggests putative constructions shown in Figure S4. Characterisation of the lipids that did not require additional MS^n fragmentation was based on the elemental composition prediction and supplemented by the specific retention times corresponding to the lipid classes (see Table S1). Without considering the number of isomers, 403 species of lipid were putatively annotated in different classes and used for the statistical analysis given in the main text. More lipid species were detected for PC and PS than for other polar lipids.

Table S1. Predominant lipids in giant panda milk

Main class	Subclass	Number of species detected	Retention time range (min)	Detected ion (ESI positive/negative)	Detected ion for comparative analysis	Characteristic fragmentation (ESI positive/negative)	Example of MS^n analysis
Phosphatidylserine (PS)	Monoacyl-PS	25	11.8-12.5	M+H/M-H	M+H	M+H-(18+87+80)/M-H-87	PS (18:0/0:0)
	Diacyl-PS	38	10.8-11.5	M+H/M-H	M+H	M+H-185/M-H-87	PS (18:0/22:6)
Phosphatidylcholine (PC)	Monoacyl-PC	23	15.5-16.5	M+H/M+COOH-	M+H	M+H-(18+59)/(M+COOH-)-60	PC (18:1/0:0)
	Diacyl-PC	43	14.5-15.5	M+H/M+COOH-	M+H	M+H-59/(M+COOH-)-60	PC (16:0/16:0)
Phosphatidylinositol (PI)	Monoacyl-PI	2	5.5-6.5	$M+NH_4+$/M-H	M-H	M+NH4+-(18+259)/M-H-180	PI (20:4/0:0)
	Diacyl-PI	16	4.0-5.0	$M+NH_4+$/M-H	M-H	M+NH4+-(259+40)/M-H-180	PI (20:4/18:0)
Phosphatidylethanolamine (PE)	Monoacyl-PE	16	11.8-12.5	M+H/M-H	M-H	M+H-(18+123)/M-H-197=free fatty acid	PE (18:1/0:0)
	Diacyl-PE	19	10.5-11.5	M+H/M-H	M-H	M+H-141/two free fatty acids	PE (18:1/16:0)
Sphingomyelin (SM)		27	15-16	M+H/M+COOH-	M+H	M+H-(18+59)/(M+COOH-)-60	SM (d18:1/24:1)
Glycerolipids (GL)		49	2.0-3.0	$M+NH_4+$	M+NH4+	M+H	TAG (52:6)
Free fatty acid (FFA)		23	2.5-3.5	M-H	M-H	M-H-44	DHA (22:6)
FFA derivatives		65	3.0-6.0	$M+NH_4+$/M-H	N/A		
Sterols		14	2.0-3.0	$M+NH_4+$	N/A		
Others annotated in Lipid MAPS		43	N/A				
**Total**		**403**					

N.B. Based on accurate mass searching (± 3ppm), the glycerolphospholipids were putatively annotated and classified by using of glycerolphospholipid prediction routine within MZMine 2.10. The other lipids were putatively annotated and classified by interrogating the Lipid MAPS database. Reliable MS^n fragment data were only obtained on the lipids with sufficient intensity of LC-MS signal at MS level 1. An example of MS^n analysis for each lipid class and subclass is shown in Figure S3.

ESI = electro spray ionisation; TAG = triacylglycerol; DHA = docosahexaenoic acid

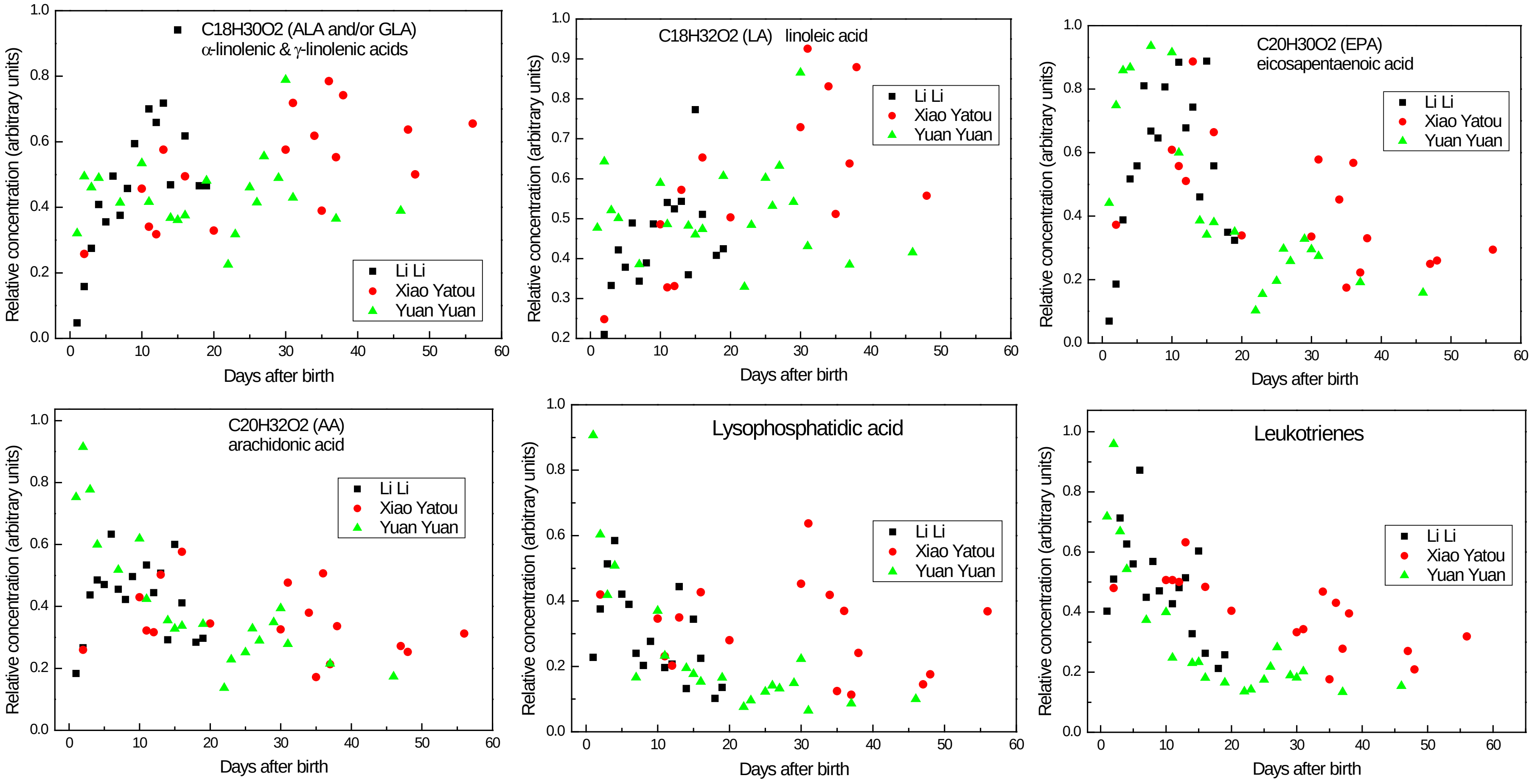

Figure S1. Time courses for selected lipids in the milk of three giant pandas with time after birth. Additional to those dealt with in the main article text. ALA and GLA are indistinguishable in the LC-MS analytical system used. Only α-linolenic and linoleic acids are considered to be essential for humans, and are precursors for the conditionally essential fatty acids and their derivatives. The leukotrienes plot refers to the non-cysteinyl forms that may include LTA_4 and LTB_4.

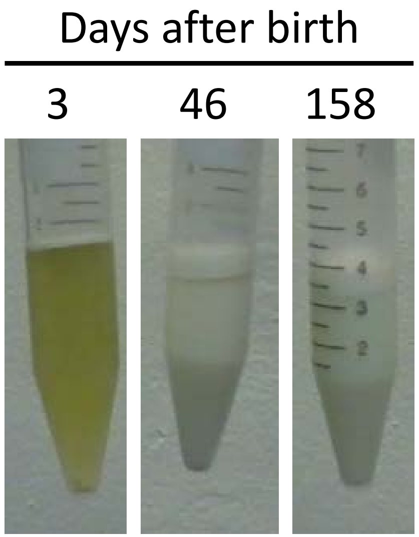

Figure S2 – Changes in appearance of giant panda milk with time, illustrating the changes in colour and fat layer depth. Approximately 4ml samples of milk from giant panda Shu Qing taken on the indicated days post-partum were centrifuged for 15 minutes at 4,500 r.p.m in a bench centrifuge at 4°C.

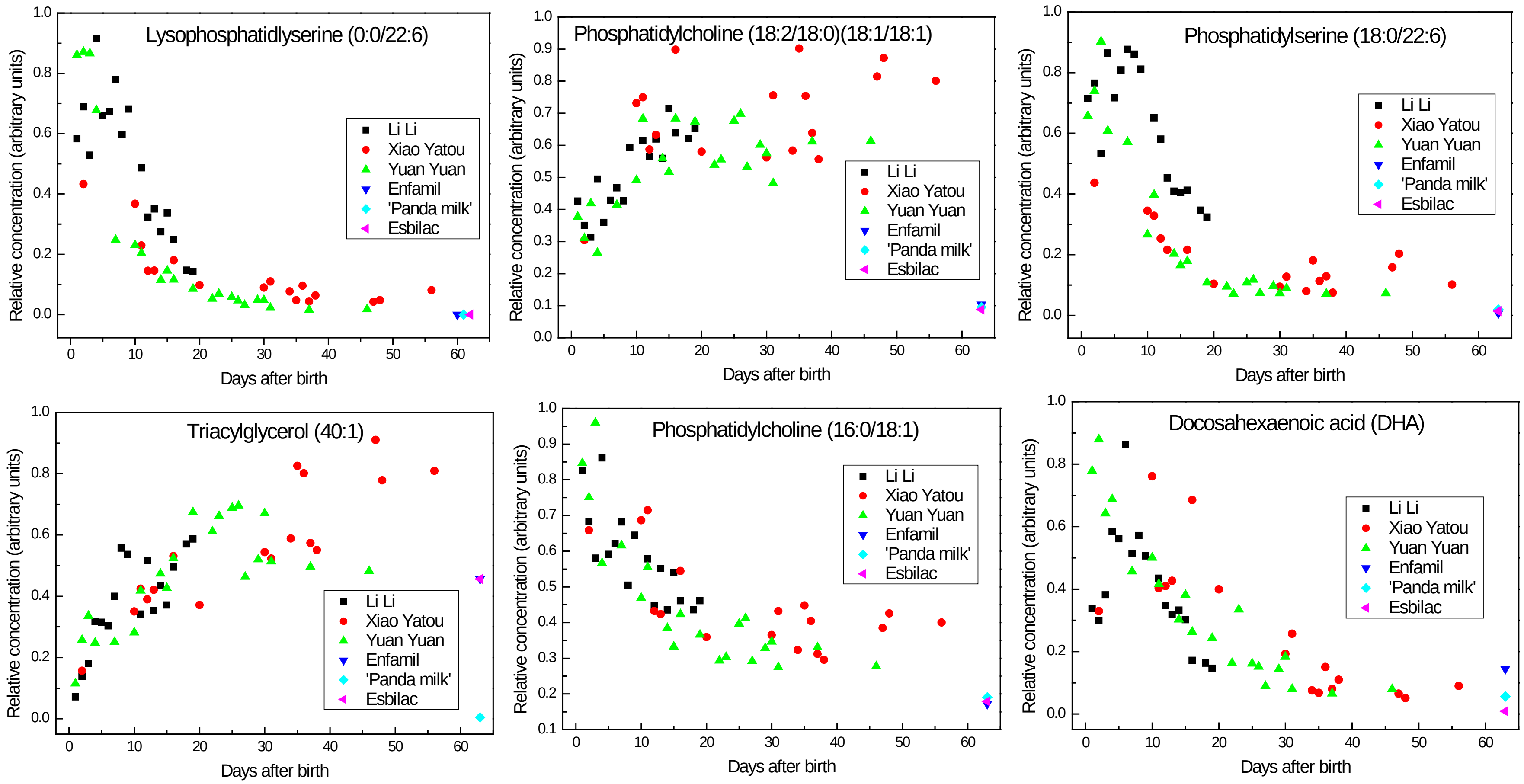

Figure S3. Comparison between the time-dependent lipid profiles of giant panda milk with lipid content of artificial milk replacers. These graphs are the same as in Figure 1 of the main text but here include data for three milk replacers used for giant panda cubs given to the right of each panel. Powdered human infant formula (®Enfamil, Mead Johnson, Indiana, USA) and puppy milk replacer (®Esbilac, PetAg, Illinois, USA) were obtained from commercial sources. These formulations are currently used directly or as a base for milk substitutes for giant panda cubs. A panda milk replacer (henceforth "Panda milk") formulated for research use but now proposed for application, was obtained from the manufacturer (Morinyu Sunworld

Co., Tokyo, Japan) and described in reference [2]. This specialist replacer contains little or none of the lipids we find natural to giant panda colostrum (this study), and is also known to contain levels of lactose in excess of those occurring in natural panda milks after day 10 post-partum [1, 3].

Figure S4. Mass spectra of the main lipids identified in giant panda milk. The first frame is an example of serial mass spectrometry analysis used here in the identification phosphatidylserine, followed by MS, MS^2 and MS^3 spectra of the main lipids identified in giant panda milk. Abbreviations - PS, phosphatidylserine; PC, phosphatidylcholine; PI, phosphatidylinositol; PE, phosphatidylethanolamine; SM, sphingomyelin; TAG, triacylglycerol; DHA, docosahexaenoic acid.

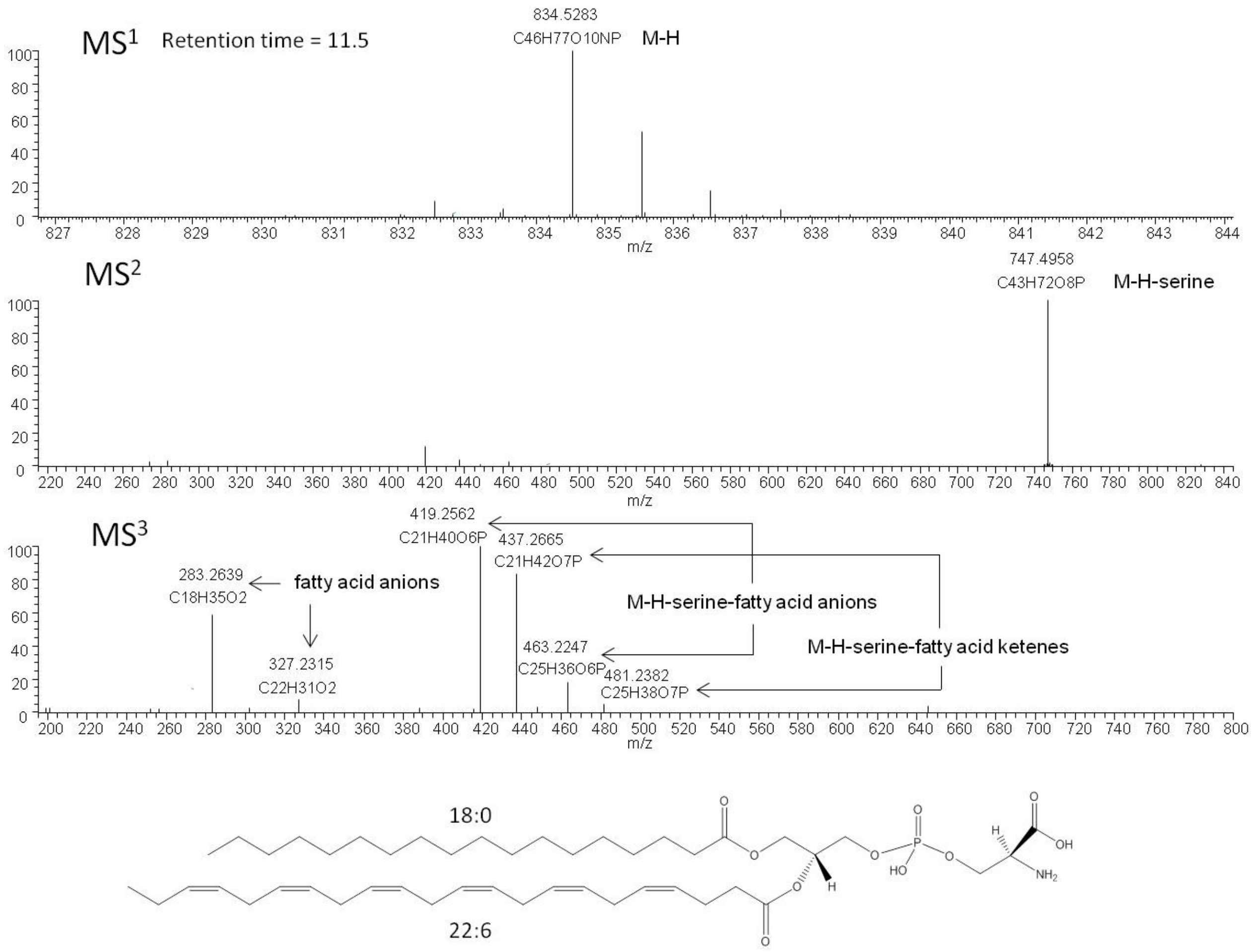

PS (18:0/0:0)

F:\Alex\...\Colostrum-Pos-general-2 04/11/2015 21:57:20

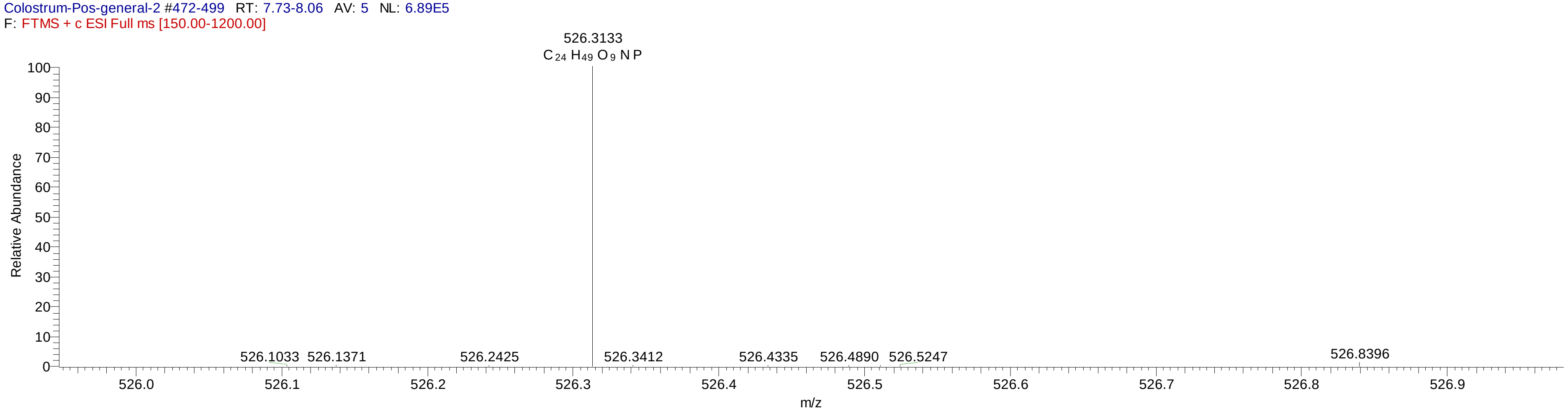

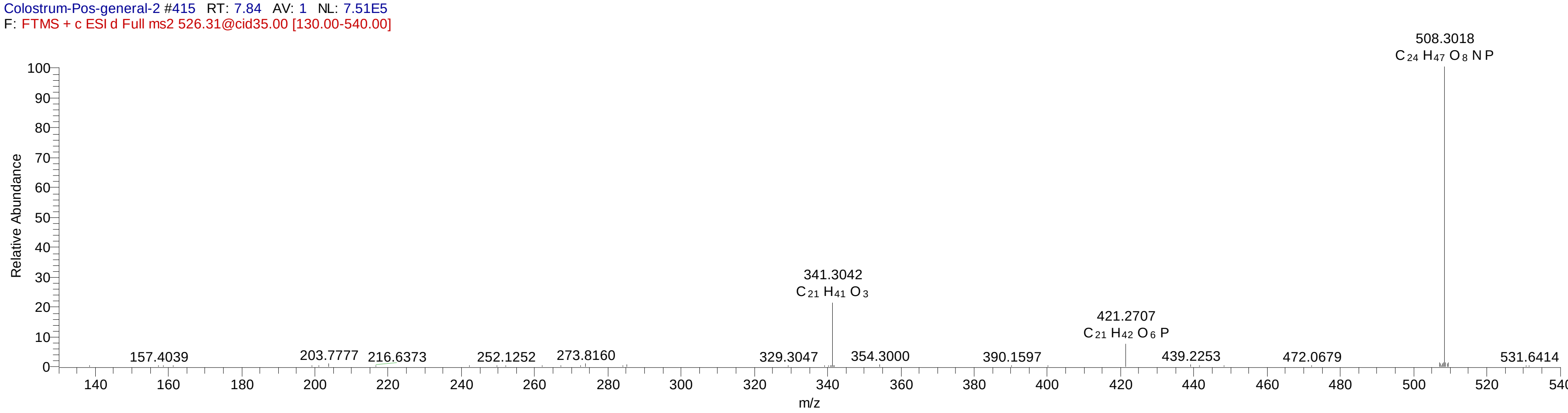

PS (18:0/22:6)

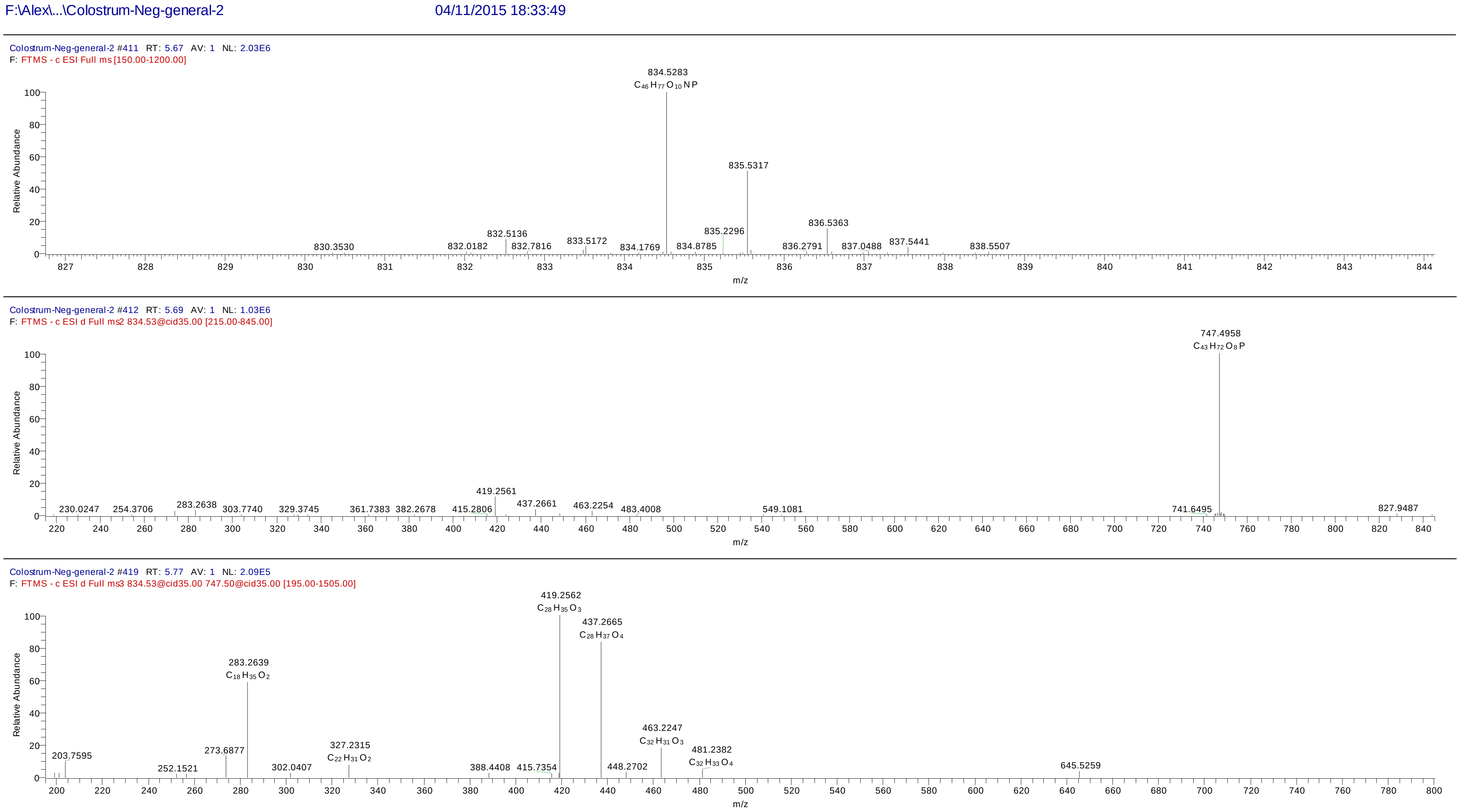
F:\Alex\...\Colostrum-Neg-general-2
04/11/2015 18:33:49
Colostrum-Neg-general-2 #411 RT: 5.67 AV: 1 NL: 2.03E6
F: FTMS - c ESI Full ms [150.00-1200.00]
834.5283
C46 H77 O10 N P
835.5317
836.5363
832.5136
835.2296
833.5172
837.5441
830.3530
832.0182
832.7816
834.1769
834.8785
836.2791
837.0488
838.5507
Relative Abundance
m/z
Colostrum-Neg-general-2 #412 RT: 5.69 AV: 1 NL: 1.03E6
F: FTMS - c ESI d Full ms2 834.53@cid35.00 [215.00-845.00]
747.4958
C43 H72 O8 P
419.2561
437.2661
283.2638
463.2254
230.0247
254.3706
303.7740
329.3745
361.7383
382.2678
415.2806
483.4008
549.1081
741.6495
827.9487
Relative Abundance
m/z
Colostrum-Neg-general-2 #419 RT: 5.77 AV: 1 NL: 2.09E5
F: FTMS - c ESI d Full ms3 834.53@cid35.00 747.50@cid35.00 [195.00-1505.00]
419.2562
C28 H35 O3
437.2665
C28 H37 O4
283.2639
C18 H35 O2
463.2247
C32 H31 O3
481.2382
C32 H33 O4
327.2315
C22 H31 O2
273.6877
203.7595
252.1521
302.0407
388.4408
415.7354
448.2702
645.5259
Relative Abundance
m/z

PC (18:1/0:0)

F:\Alex\...\Colostrum-Neg-general-2 04/11/2015 18:33:49

Colostrum-Neg-general-2 #1291 RT: 17.86 AV: 1 NL: 3.68E6
F: FTMS - c ESI Full ms [150.00-1200.00]

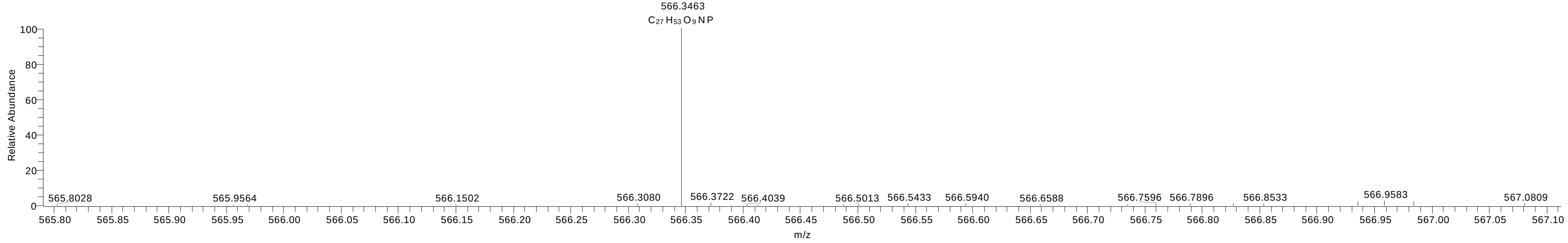

Colostrum-Neg-general-2 #1116 RT: 17.51 AV: 1 NL: 6.76E5
F: FTMS - c ESI d Full ms2 566.35@cid35.00 [145.00-1145.00]

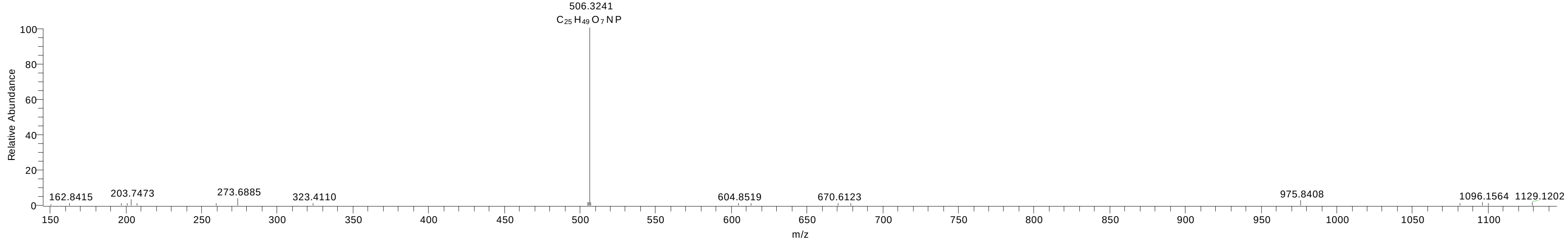

Colostrum-Neg-general-2 #1265 RT: 17.53 AV: 1 NL: 3.93E5
F: FTMS - c ESI d Full ms3 566.35@cid35.00 506.32@cid35.00 [125.00-1025.00]

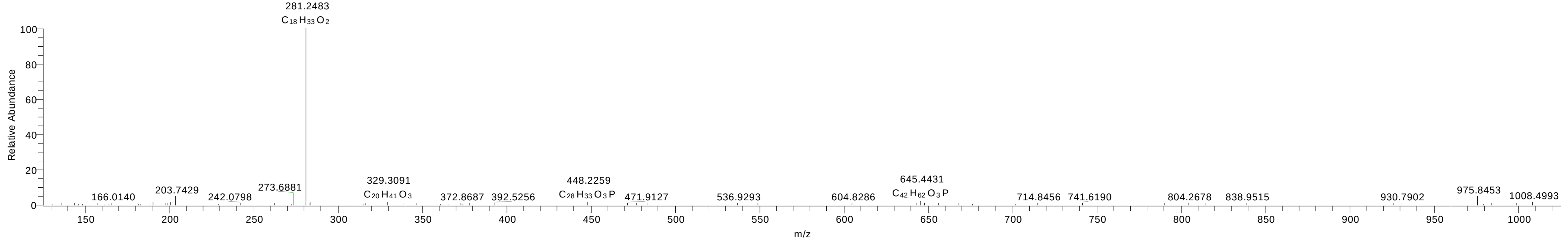

PC (16:0/16:0) The PC lipids in negative ion mode are in the form of their formic acid adducts. MS^2 removes formic acid plus a methyl group, and MS^3 gives the fatty acids on the glycerol backbone. In this case, only the negative ion for palmitic acid at m/z 255.2330 can be seen.

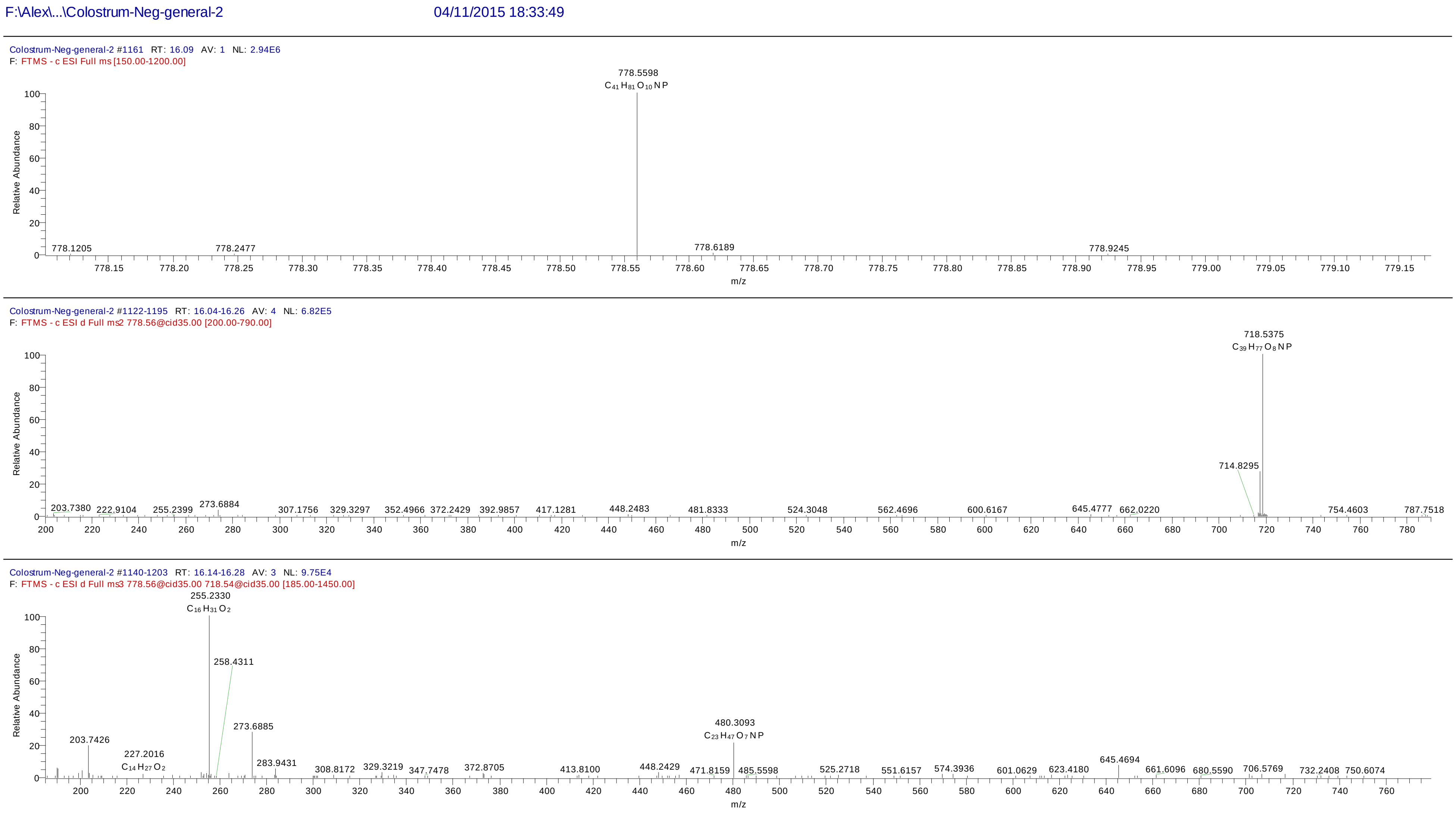

PI (20:4/0:0) In this lyso PI lipid, the negative ion at 303.2327 corresponding to tetracosanoic acid can be seen. The ion at m/z 315.0486 corresponds to the glycerol backbone with phosphoinositol attached following the loss of C20:4.

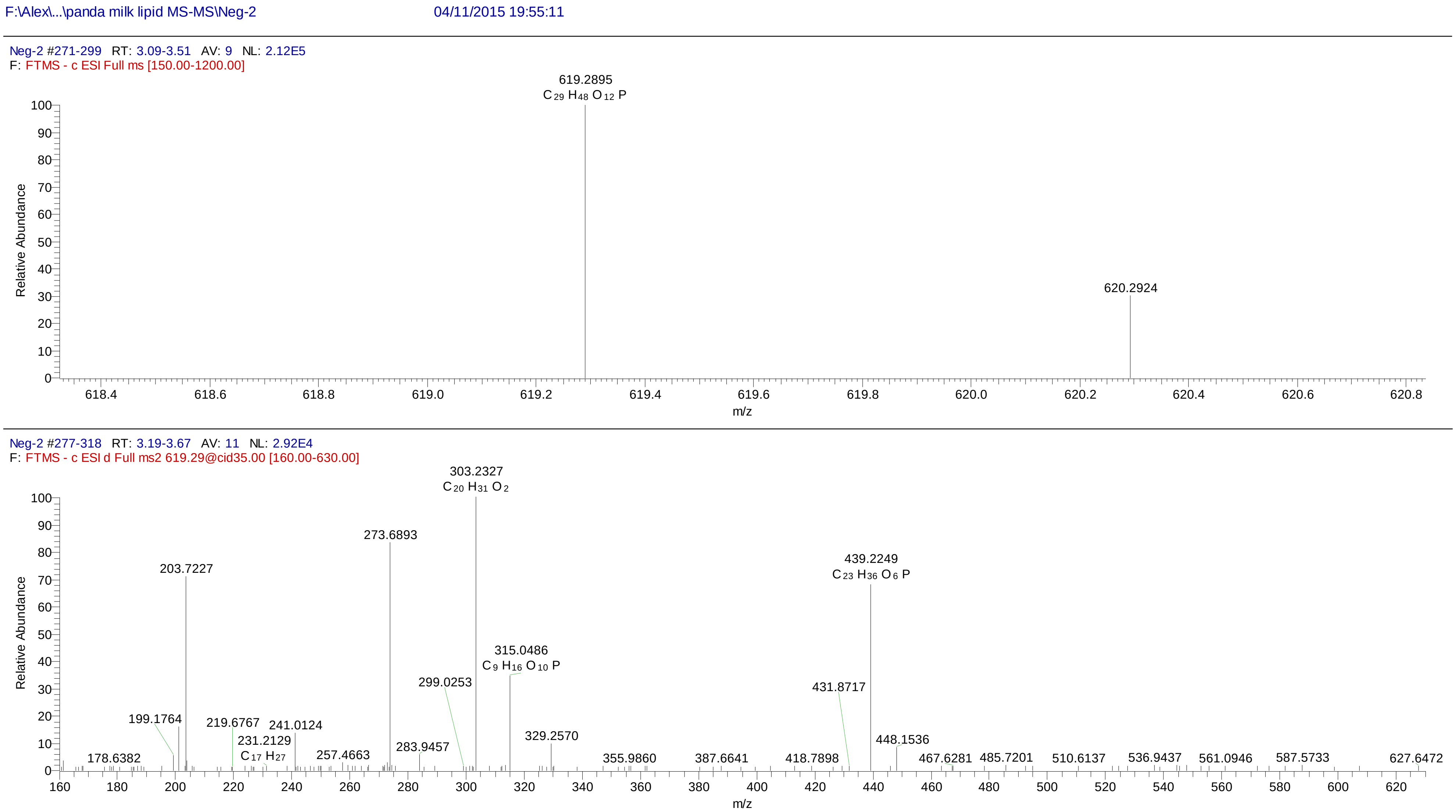

PI (20:4/18:0) The MS[2] spectrum shows ions at 283.2645 for C18:0 and at 303.2332 for C20:4. The ion at 588.3096 results from the loss of C20:4 from the molecular ion. There is no evidence of an alternative substitution pattern. MS[3] carried out on 581.3096 produces an ion at 419.2571 due to loss of the inositol. The ion at m/z 297.0387 is due to loss of C18:0 and the ion at 283.2645 is due to the negative ion of C18:0. The ion at m/z 223.0016 is due to inositol phosphate - 2xH_2O.

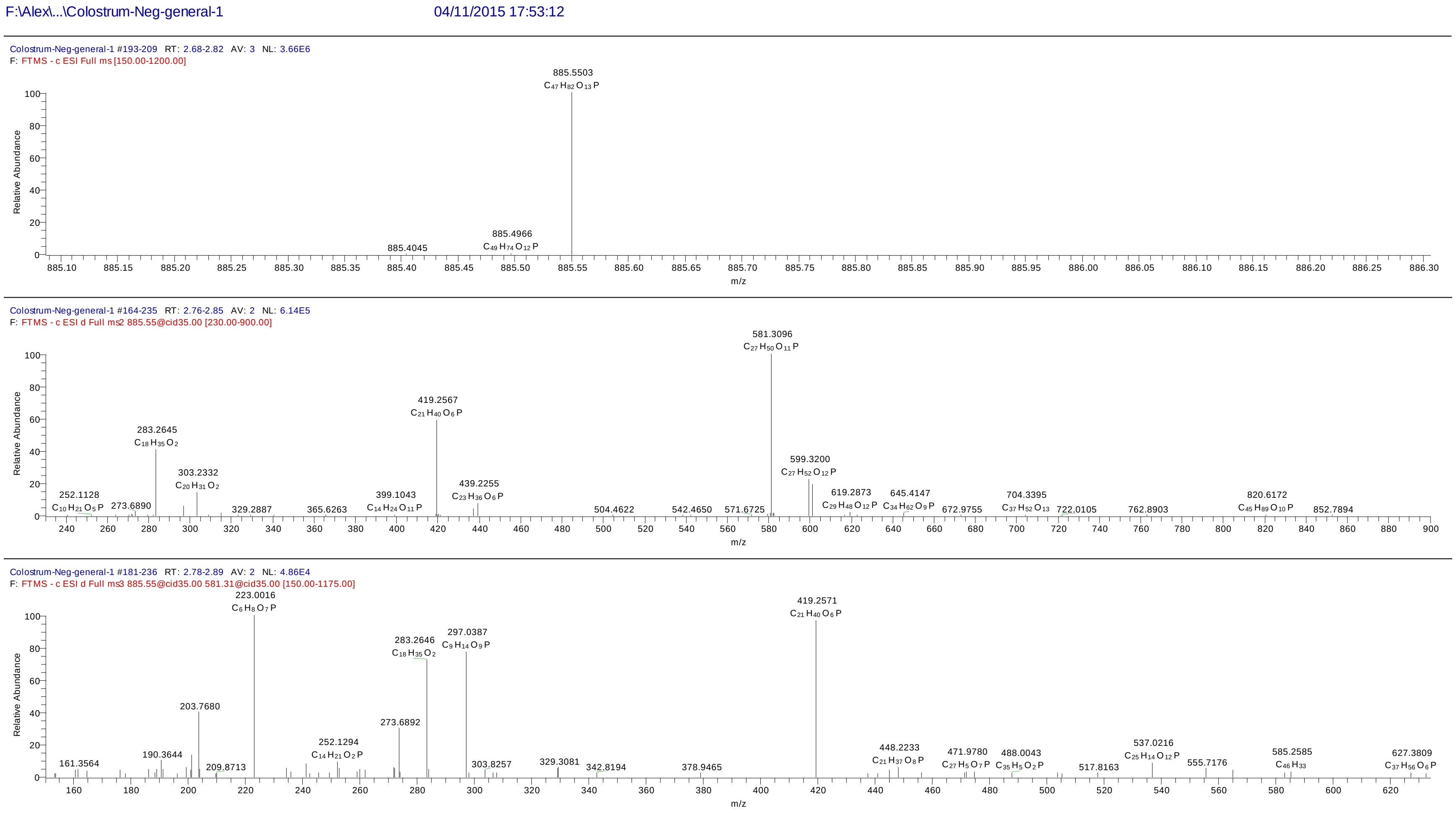

PE (18:1/0:0)

F:\Alex\...\Colostrum-Pos-general-3 04/11/2015 22:38:02

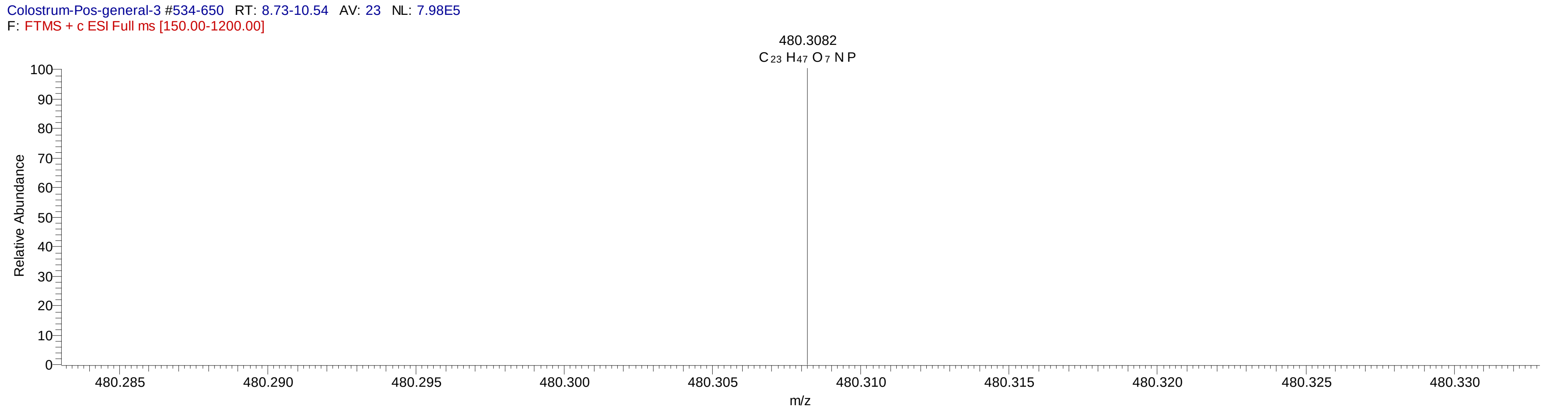

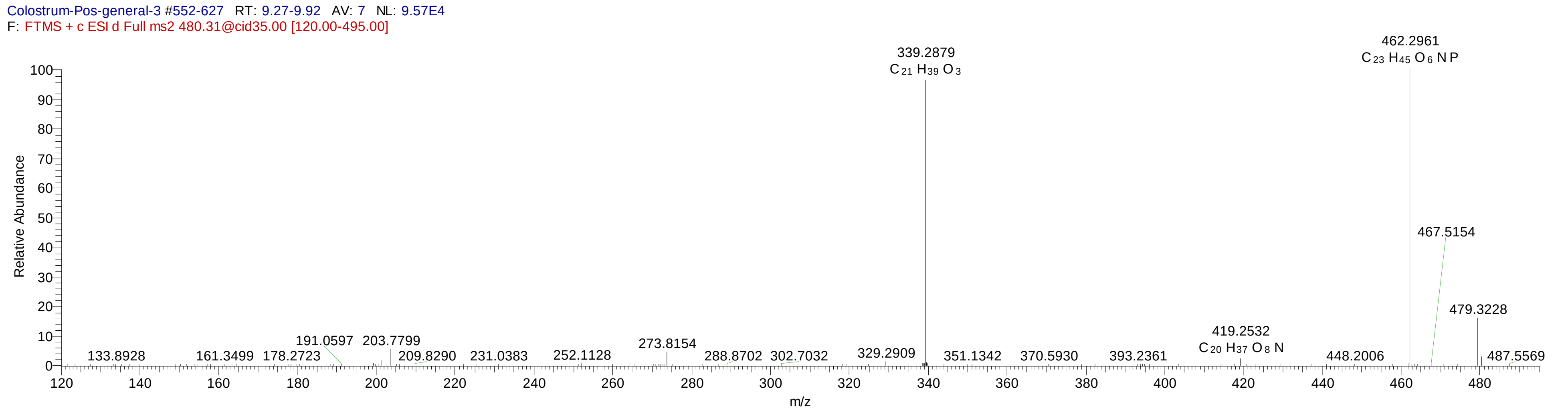

PE (18:1/16:0)/(18:0/16:1) The ions at m/z 253.2176, 255.2338, 281.2493 and 283.2664 corresponding in C16:1, C16:0, C18:1 and C18:0 indicate that this peak comprises two isomers with different fatty acid chains.

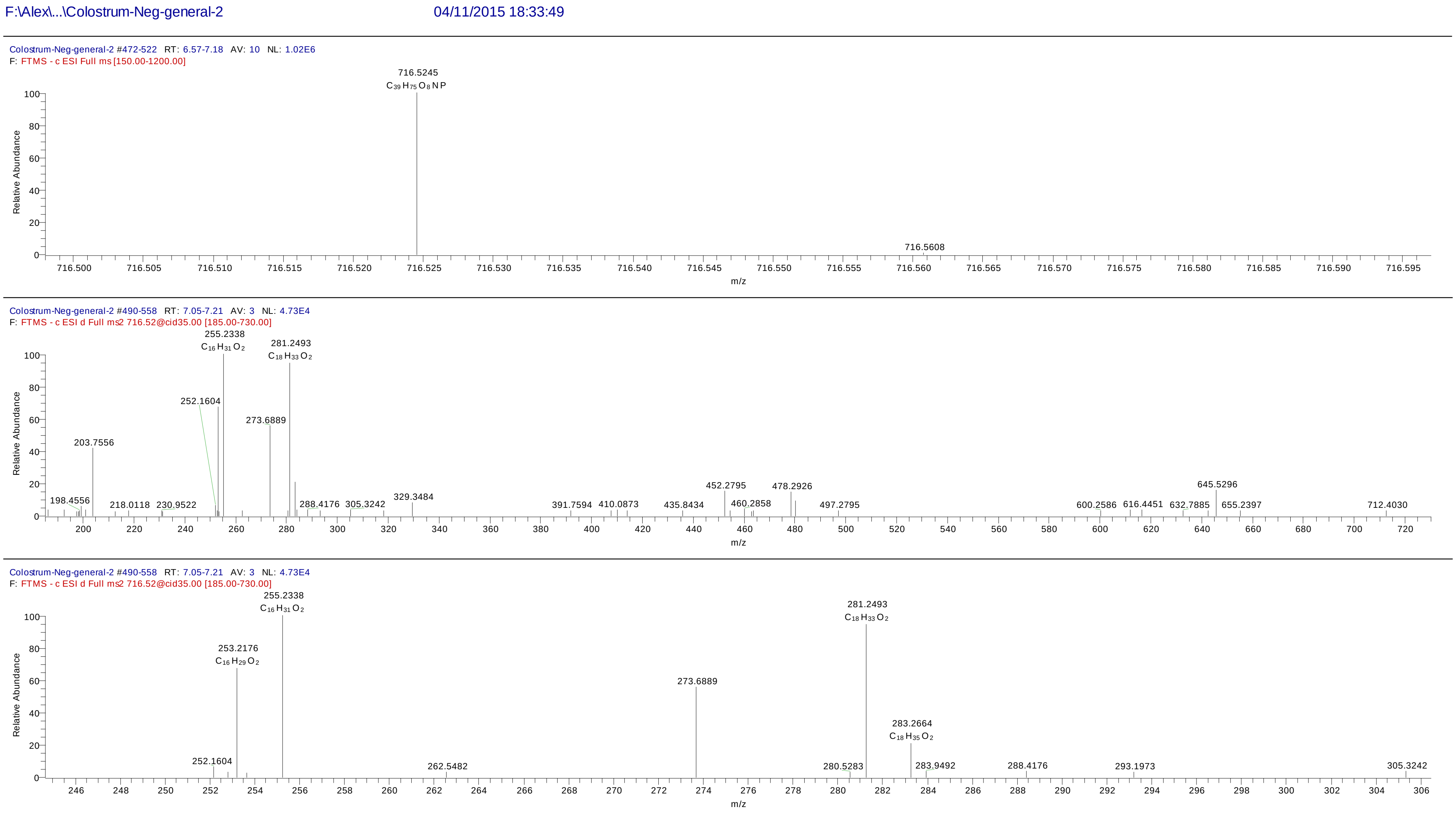

SM (d18:1/16:0) The formula is consistent with a phytosphingolipid which has an additional hydroxyl group in comparison with most mammalian sphingolipids. The additional group may be acetylated since the ion at m/z 687.5437 results from the loss of acetic acid. The MS^3 spectrum for the ion at 687.5437 produces a fragment at m/z 616.4716 which must result from the loss of the trimethyl ammonium head group and an extra carbon. The most diagnostic fragment is at m/z 449.3163 which results from the loss of palmitoyl from the amine functionality of the sphingolipid.

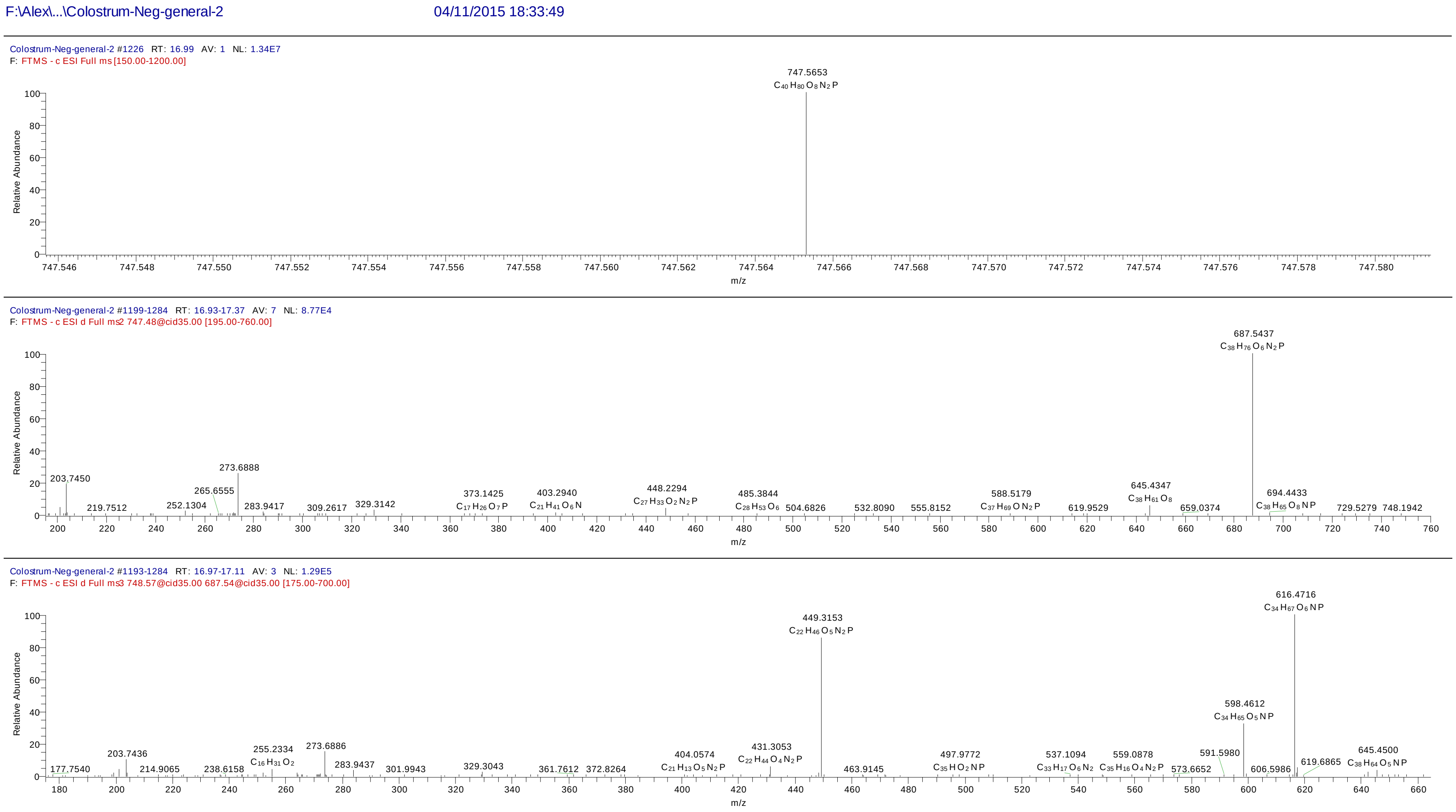

TAG (52:6) Main fatty acids C20:5, C22:6, C16:1, C18:4

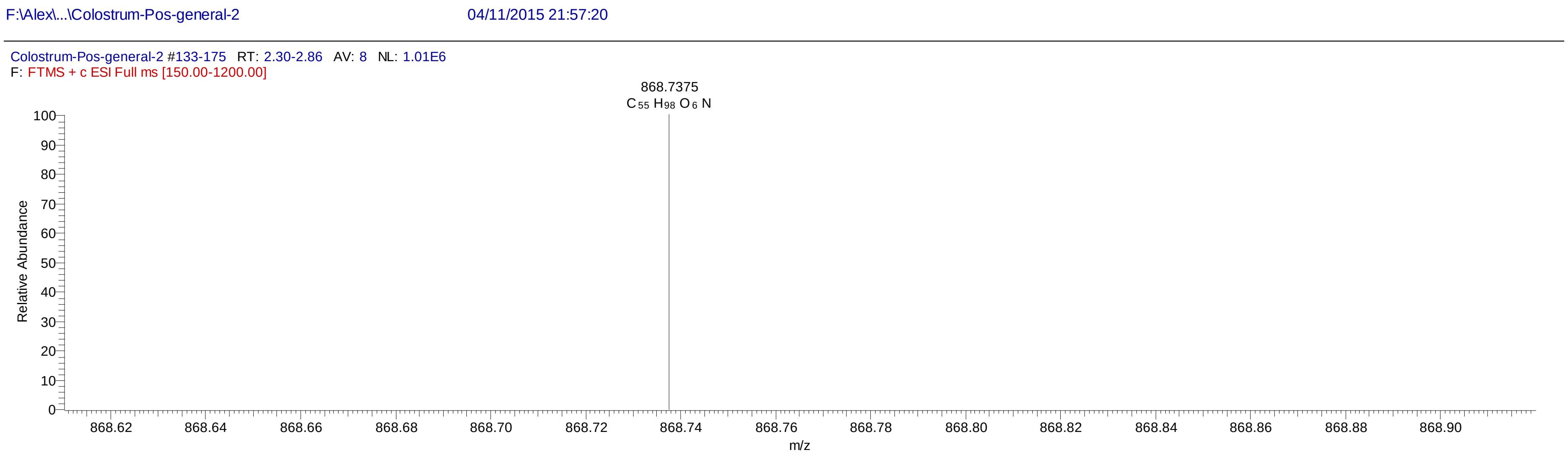

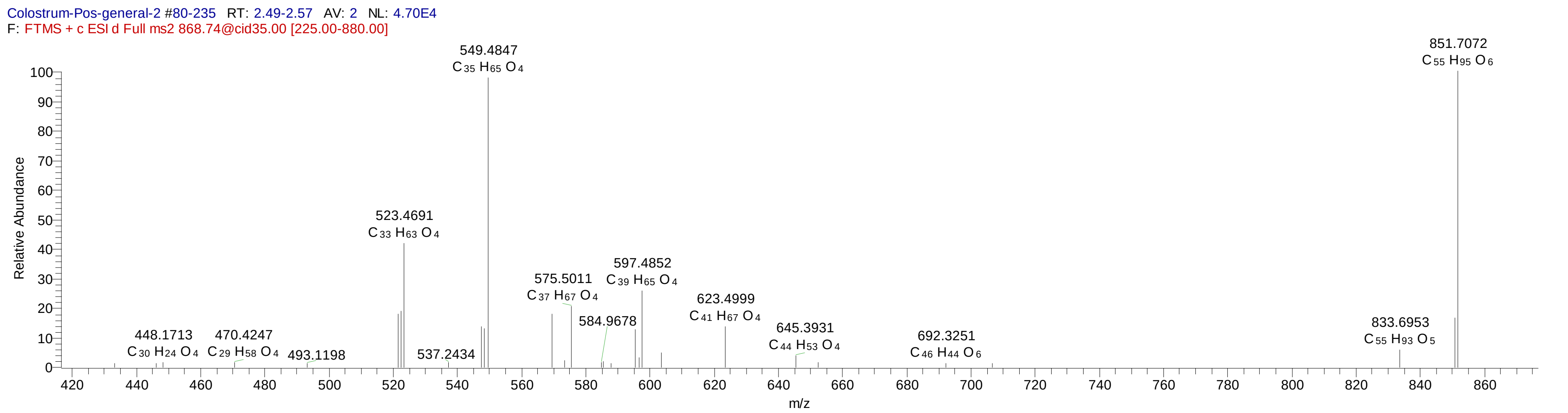

DHA (22:6) The MS^2 spectrum of DHA shows loss of CO_2 to yield the ion at m/z 283.2430.

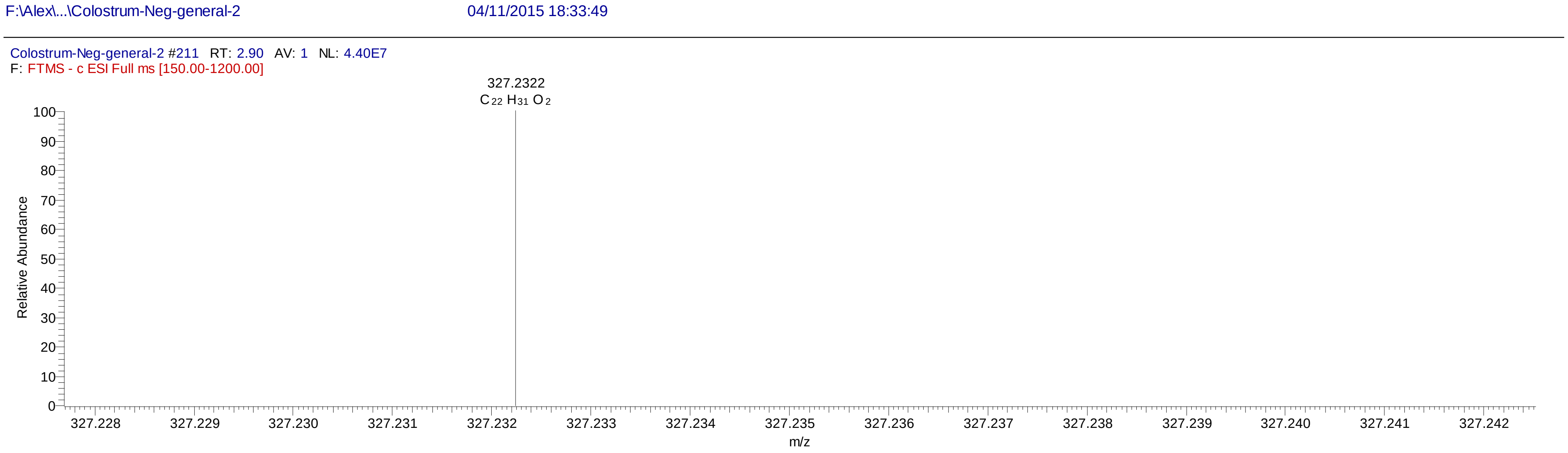

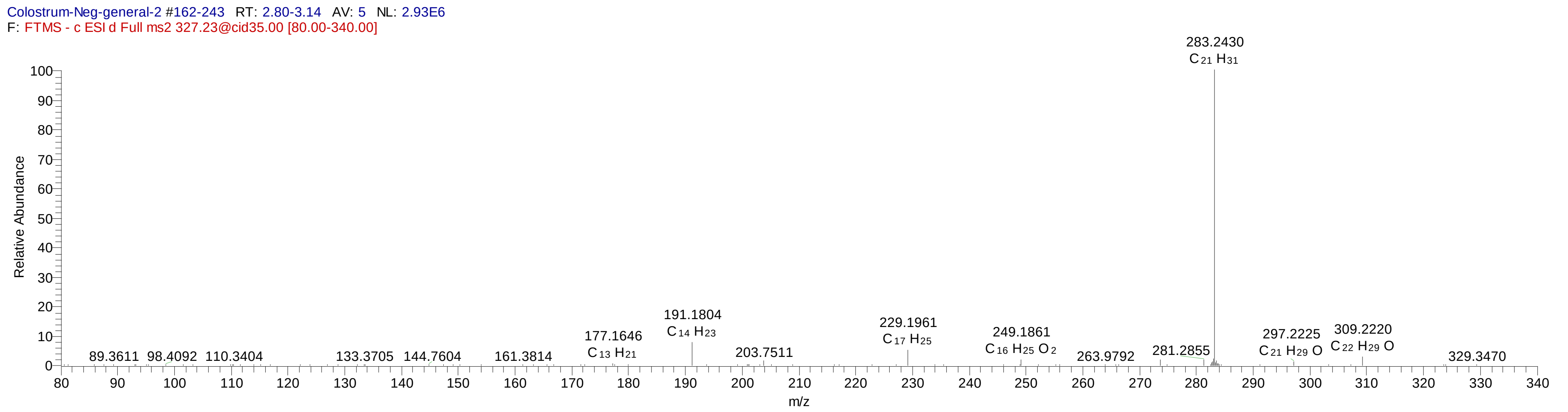

Table S2. Reproductive histories and milk sampling times of giant pandas from whom samples were collected. All mothers and cubs were born in captivity. Giant panda international studbook numbers as given. Parturition dates in bold indicate the beginning of the lactation period from which samples were collected. See also ref. [1].

**Panda mother information**	**Date of milk collection**	**Postpartum day**
Name: Li Li<br>Studbook No. 387<br>Age: 20 years<br>Parturition date: 28 July 2012<br>Lactation history:<br>12 July 2001, twins. died 19 Sept & 17 Oct, 2001<br>08 July 2002, single, died 08 July 2002<br>03 Aug 2007*, single, died same day<br>19 July 2009, twins<br>24 July 2011, single<br>28 July 2012, twins, 1 died same day<br>Total 17 samples	29 Jul 2012	1
	30 Jul 2012	2
	31 Jul 2012	3
	01 Aug 2012	4
	02 Aug 2012	5
	03 Aug 2012	6
	04 Aug 2012	7
	05 Aug 2012	8
	06 Aug 2012	9
	08 Aug 2012	11
	09 Aug 2012	12
	10 Aug 2012	13
	11 Aug 2012	14
	12 Aug 2012	15
	13 Aug 2012	16
	15 Aug 2012	18
	16 Aug 2012	19
Name: Yuan Yuan<br>Studbook No. 561<br>Parturition date: 25 August 2012<br>Age: 9 years<br>Lactation history:<br>06 Sept 2008, single<br>15 Aug 2010, twins<br>25 Aug 2012, twins, 1 died same day<br>Total 21 samples	26 Aug 2012	1
	27 Aug 2012	2
	28 Aug 2012	3
	29 Aug 2012	4
	01 Sep 2012	7
	04 Sep 2012	10
	05 Sep 2012	11
	08 Sep 2012	14
	09 Sep 2012	15
	10 Sep 2012	16
	13 Sep 2012	19
	16 Sep 2012	22
	17 Sep 2012	23
	19 Sep 2012	25
	20 Sep 2012	26
	21 Sep 2012	27
	23 Sep 2012	29
	24 Sep 2012	30
	25 Sep 2012	31
	01 Oct 2012	37
	10 Oct 2012	46
Name: Xiao YaTou<br>Studbook No. 635<br>Age: 6 years<br>Parturition date: 14 August 2012<br>Lactation history: this was the first parturition	16 Aug 2012	2
	24 Aug 2012	10
	25 Aug 2012	11
	26 Aug 2012	12
	27 Aug 2012	13
	30 Aug 2012	16

Panda mother information	Date of milk collection	Postpartum day
17 samples	03 Sep 2012	20
	13 Sep 2012	30
	14 Sep 2012	31
	17 Sep 2012	34
	18 Sep 2012	35
	19 Sep 2012	36
	20 Sep 2012	37
	21 Sep 2012	38
	30 Sep 2012	47
	01 Oct 2012	48
	09 Oct 2012	56

References for Supplementary Information

1. Zhang, T., et al., *Changes in the milk metabolome of the giant panda (Ailuropoda melanoleuca) with time after birth - three phases in early lactation and progressive individual differences.* Plos One, 2015. **10**(12): p. e0143417.
2. Zhang, Z., et al., *Analysis of the breast milk of giant pandas (Ailuropoda melanoleuca) and the preparation of substitutes.* Journal of Veterinary Medical Science, 2016. **78**(5): p. 747-754.
3. Griffiths, K., et al., *Prolonged transition time between colostrum and mature milk in a bear, the giant panda, Ailuropoda melanoleuca.* Royal Society Open Science, 2015. **2**(10): p. 150395.
